# P49 Improving rates of IV to oral antibiotic switch, an education and training approach

**DOI:** 10.1093/jacamr/dlaf118.056

**Published:** 2025-07-14

**Authors:** Amirah Hussain, Laura Ahearn, Amina Deria, Rachel Hemingway

**Affiliations:** Northern Care Alliance, UK; Northern Care Alliance, UK; Northern Care Alliance, UK; Northern Care Alliance, UK

## Abstract

**Objectives:**

To reduce IV antibiotic use by 10% across the medical wards at an acute teaching hospital between May and July 2024.

**Background:**

Improving IV to oral antimicrobial switch (IVOS) rates facilitates patient flow, reduces nursing time spent reconstituting IV antibiotics, increases the use of cost-effective antibiotics, reduces the ward level carbon footprint, and decreases the risk of developing line related infections. The project aimed to reduce IV antimicrobial use across medical wards through the training of champions to implement an ABCDE protocol sticker. The protocol was developed from the ‘Antimicrobial IV-to-oral switch: criteria for prompt switch’ (gov.uk).^1^

**Methods:**

A small-scale test of change showed that the ABCDE protocol increased the rate of IVOS by 50% on a respiratory ward. This project set out to replicate that success across all medical wards. To ensure success of the project at least one nurse and doctor champions were identified on each of the medical wards, and a ‘train the trainer’ approach was adopted. Training materials were developed for all champions and education sessions were held to empower them to cascade the teaching to nursing and clinical staff on the target wards. Throughout the project our champions ensured there was ongoing engagement at ward level throughout the months of implementation of the test of change. To maintain motivation, updates on progress were circulated to the teams.

**Results:**

Although the IV antibiotic use was not reduced by 10%, there was an overall reduction in the use of IV antibiotic usage. Over the 3-month period 555 antibiotic doses were avoided saving 185 nursing hours that would have been spent preparing IV antibiotics. There was also a saving of £395 on antibiotics, £1876 on consumables, a saving of 108/3kgCO2 and £117.71 on high temperature waste. The project also trained 15 nurse and doctor champions and the resident doctor cohort on when to appropriately switch from IV to oral antibiotics. Pre- and post-teaching questionnaires highlighted that nursing staff felt empowered by the protocol, and medical staff felt the prompt was a useful reminder to review antibiotics. We are also influencing national best practice, with the ABCDE protocol having been included in national evidence bundles for other trusts to utilize.

**Conclusions:**

Antimicrobial stewardship (AMS) interventions are even more powerful when involving the multi-disciplinary team. The ABCDE protocol empowered staff groups to take a proactive role in the reviewing of IV antibiotics.
